# Threat, efficacy, and the ambivalent role of COVID-19 news: an EPPM analysis of health discrimination and preventive behaviors

**DOI:** 10.3389/fpubh.2026.1790676

**Published:** 2026-04-02

**Authors:** Liangying Ma, Gege Fang, Zhengxin Liu

**Affiliations:** 1 School of Public Administration, Central South University, Changsha, China; 2 School of Digital Media and Design Arts, Beijing University of Posts and Telecommunications, Beijing, China; 3 Beijing Key Laboratory of Network System and Network Culture, Beijing University of Posts and Telecommunications, Beijing, China; 4 School of Journalism and Communication, Tsinghua University, Beijing, China

**Keywords:** COVID-19, Extended Parallel Process Model, fear, health discrimination, news

## Abstract

**Introduction:**

During global public health crises, news media play a crucial role in disseminating information about prevention, treatment, and recovery. This study aims to investigate the social and psychological impact of news during the prevention and control process of COVID-19 in China.

**Methods:**

We collected data from 598 participants and employed the Extended Parallel Process Model (EPPM) to analyze the influence of different combinations of perceived threat and perceived efficacy in news on fear, compliance with preventive measures, and health discrimination.

**Results:**

The findings reveal significant associations between perceived threat and various outcomes: increased fear, enhanced compliance with preventive measures, and heightened health discrimination. In this process, perceived efficacy has a significant moderating effect: under high perceived efficacy conditions, fear's promotion of health discrimination is suppressed, and its encouragement of preventive measures is enhanced.

**Conclusion:**

This study highlights that balanced health communication in media, conveying both threats and efficacy, is crucial for mitigating health discrimination and sustaining public motivation for preventive behaviors.

## 1 Introduction

In late 2022, China underwent a pivotal shift in its pandemic management strategy, transitioning from the stringent “dynamic zero-COVID” policy to a framework centered on individual responsibility. As centralized systemic control concluded, the incidence of COVID-19 infections increased dramatically. Guided by the social ethos of “being the primary guardian of one's own health” (1), the responsibility for risk mitigation devolved from the state to individual citizens. In this volatile transition, news media played a dual role: while disseminating essential preventive knowledge, it also saturated the public sphere with high-threat messaging emphasizing the severity of and susceptibility to the virus (2, 3). Paradoxically, the persistent stigmatization and discrimination against those infected with or recovered from COVID-19 did not attenuate with the easing of policy. Despite governmental interventions to curb employment bias and social exclusion (4), infected and recovered individuals continued to face institutional barriers, unfair treatment in the workplace, and online harassment, highlighting a profound tension between public health mobilization and social solidarity.

While rooted in evolutionary adaptation, health discrimination paradoxically undermines protective efforts and generates systemic risks in modern society (5). Given that such discrimination impulses often endure well beyond the acute phase of a pandemic, they require deliberate strategic intervention (6). While existing research has extensively employed the Extended Parallel Process Model (EPPM) to understand how individuals process such risk information, scholarly attention has pre-dominantly focused on adaptive health behaviors, such as vaccination intentions and compliance with hygiene protocols (7–9). These studies demonstrate that communication strategies aligned with EPPM effectively drive self-protection. Yet, a critical concern remains: how to encourage protective behaviors without triggering destructive social exclusion. Current empirical evidence in this field fails to adequately elucidate how the same cognitive appraisals that motivate compliance might simultaneously fuel health discrimination. Although EPPM traditionally frames efficacy as a driver for danger control, its potential role in mitigating the stigma and discrimination responses remains under-explored.

This research revisits the traditional pathways of the EPPM to account for the broader discourse of health discrimination. We posit that the prevailing reliance on fear appeals in news media may function as a double-edged sword: while effectively increasing compliance with preventive measures, it may facilitate a “us vs. them” narrative that rationalizes discrimination as a defensive psychological response. To empirically examine this mechanism, we conducted a 2 × 2 experiment during the critical juncture of China's policy transition in early 2023. This study extends the EPPM by introducing health discrimination as a key social outcome, complementing traditional individual level behavioral metrics. Furthermore, by examining the moderating role of perceived efficacy, this research illustrates how news framing can be optimized to encourage public compliance without incurring the social cost of marginalizing vulnerable populations. These insights offer enduring value for post-pandemic risk management, providing an alternative approach for maintaining social inclusivity and resilience during future public health emergencies.

## 2 Literature review

### 2.1 Extended parallel process model (EPPM)

#### 2.1.1 Theoretical foundations of the EPPM

The Extended Parallel Process Model (EPPM) stands as a widely recognized theoretical framework for elucidating the mechanisms by which individuals process health-risk information and for optimizing communication strategies to foster protective behaviors (10). The theoretical lineage of the EPPM traces back to Leventhal's 1970 Parallel Response Model, a seminal contribution to fear appeal research that proposed two distinct pathways for processing threat: danger control and fear control. Rogers (11) further refined it through Protection Motivation Theory, identifying four elements associated with fear appeals: susceptibility, severity, response efficacy, and self-efficacy. These four elements drive two cognitive mediators: threat appraisal and coping appraisal. Specifically, susceptibility and severity trigger threat appraisal, while response efficacy and self-efficacy drive coping appraisal (12). When both appraisals are positive, the audience is motivated to engage in protective actions, leading to attitudinal or behavioral changes; conversely, if one appraisal is negative, the audience may react adversely.

Synthesizing these perspectives, Witte (13) formalized the EPPM, which remains the pre-dominant framework for understanding health information and predicting protective behaviors induced by messages. The EPPM posits that responses to health threats hinge on two assessment processes: threat appraisal and efficacy appraisal (14). During threat appraisal, individuals evaluate factors related to the health threat, including perceived severity and perceived susceptibility. Efficacy appraisal involves beliefs about the effectiveness of coping measures in reducing health threats (response efficacy) and the conviction in successfully adopting these measures (self-efficacy) (15). According to the EPPM, when individuals assess a health threat as harmless or irrelevant, such content does not evoke fear and does not give rise to fear appeals (13). If the perceived threat is too low, it fails to trigger the second appraisal of efficacy, and there is no motivation for further information processing. If the perceived threat crosses a critical threshold, fear is induced (16). Crucially, the outcome depends on the interaction between threat and efficacy: when both perceived threat and efficacy are high, individuals engage in proactive cognitive and behavioral actions to mitigate the health risk.

Fear appeals have historically proven effective in health communication (17, 18), such as addressing diverse public health issues, such as smoking cessation (19), vaccine hesitancy (20), and mental disorders (21). Furthermore, while scrutinizing fear's broader psychological and social externalities, scholars have noted that fear appeals may diminish in persuasive effectiveness under certain emotional states, such as anger (22). Critiques suggest that prior research may have overestimated the effectiveness of fear appeals compared to other interventions and overlooked specific destructive responses (23, 24). Beyond individual psychological defense, these control responses can manifest as significant social costs, including the exclusion and dehumanization of groups perceived as threat carriers (25, 26).

Therefore, research is increasingly shifting its focus from individual cognitive outcomes to the complex societal ramifications of public health messaging. Fear-based discourses risk perpetuating pre-judice and moralizing illness (27), which may ultimately erode community solidarity and the willingness to fulfill civic responsibilities (28). As Dimitrov et al. (29) underscore, the imperative of public health communication must extend beyond mere population control to encompass the reduction of discrimination, which is a facet historically marginalized in crisis management. The COVID-19 pandemic has renewed the urgency of tailoring communication to public needs while identifying discourses that inadvertently rationalize discrimination (30). In this context, applying the EPPM to foster social cooperation and minimize discrimination is not simply a theoretical exercise but a practical necessity for sustainable crisis management (31).

#### 2.1.2 EPPM applications in the COVID-19 context

The global COVID-19 pandemic provided a critical exigency for testing and refining the EPPM within a prolonged public health crisis. Recent studies reaffirmed the model's enduring robustness in predicting protective behaviors across geographically and culturally diverse populations (32), such as vaccination intentions, social distancing, and general compliance with preventive measures (33–36). While these studies validated the core mechanism of the model that the interplay of threat and efficacy dictates preventive outcomes, the persistent nature of the pandemic has revitalized long-standing practical concern. Most notably, when communication strategies fail to balance these components, the resulting information imbalance may render the threat less an instrument of persuasion and more a catalyst for psychological reactance or health harm (37). Consequently, scholarly inquiry has tended to examine of how specific external and internal contingencies modulate the EPPM cognitive and behavioral pathways.

Regarding external factors, the focus has evolved from media exposure to the structural distinction of the information environment. Tsoy et al. (35) found that while threat and efficacy perceptions directly influenced the intention to stay home, the participants' general media exposure did not significantly alter these core perceptions. In contrast, Chung and Jones-Jang (38) specified that the source of media matters, showing that exposure to conservative media shaped threat and efficacy perceptions, which in turn affected compliance. Furthermore, cross-national comparisons highlight significant disparities in journalistic cultures; Western news outlets often lean toward fear narratives compared to their Asian counterparts, a strategy that appeal to public discourse (39). In the contemporary digital landscape, algorithmic distribution on social media platforms tends to amplify perceived threats while simultaneously eroding efficacy, thereby destabilizing the delicate fear-efficacy equilibrium required for health promotion (40, 41).

Research has also extended the EPPM by integrating internal psychological and social variables. Scholars have examined how factors such as self-esteem can influence an individual's response to health threats (34), while other work has explored how fear functions as a key mediator between perceived risk and preventive actions (9). The crucial role of efficacy is further underscored in high-stakes professional settings; Woyessa et al. (42) found that low efficacy perceptions were a primary driver of healthcare workers' unwillingness to work during the pandemic, even when the threat was high. In regions characterized by prolonged and stringent public health mandates, research indicates that the public has increasingly succumbed to pandemic fatigue. This state of emotional and cognitive exhaustion renders conventional fear appeals ineffective, as the habitual exposure to threat messages fails to motivate further protective behavior (43).

From a practical communication standpoint, the consensus in recent literature underscores the primacy of efficacy. Studies have emphasized that targeting response efficacy is a key strategy for reducing vaccine hesitancy (44). While fear appeals capture attention, they successfully motivate protective behaviors only when paired with robust efficacy messaging that fosters a sense of agency and collective responsibility (45). Without this balance, fear appeals risk activating the fear control. Crucially, this leads to a significant field in the existing literature that need further exploration. While the EPPM has been robustly applied to explain why people comply or ignore health advice, less attention has been paid to the social collateral damage, which warrants a detailed examination to understand how health message induced threat and efficacy interact to shape not just personal compliance, but social inclusivity.

### 2.2 Health discrimination and compliance with preventive measures

Health discrimination or disease discrimination, refers to the discrimination and exclusion of individuals with certain illnesses or disabilities, also known as stigma (46). Health discrimination encompasses a multifaceted continuum of social stereotypes and pre-judices (47), ranging from individualized experiences such as internalized and felt stigma to systemic manifestations embedded in institutional practices and legislation (48). Consequently, it is conceptualized as a convergent outcome of interpersonal, intrapersonal, and environmental factors. The discrimination hinders the targeted individuals from seeking medical care, participating in healthcare, and adhering to treatments, thereby becoming a major obstacle affecting healthcare and quality of life in disease management (49). Within the Health Stigma and Discrimination Framework proposed by Stangl et al. (50), such phenomenon is conceptualized as a social process driven by specific drivers and facilitators. This Framework identifies fear of infection with communicable diseases as a driving factor that facilitates stigma “marking”, ultimately leading to health and social outcomes. This theoretical linkage provides a direct bridge to the EPPM: the threat appraisal in EPPM generates the very fear of COVID-19 that Stangl identifies as the engine of discrimination.

Despite the extensive body of research on health discrimination, a critical limitation persists regarding the perspective of contextual measurement. Existing scholarship has focused pre-dominantly on mental illnesses (51), communicable diseases such as HIV (52), and the intersectional inequalities arising from the convergence of illness with other social categorizations (53). However, these inquiries typically utilize scales designed to capture the victim's experience of perceived stigma, leaving the public's propensity to discriminate or enacted stigma insufficiently examined. The COVID-19 discrimination has the repetitive patterns of social discrimination observed in historical epidemics (54); however, it also underscores an escalating, long-term challenge exacerbated by the accelerated diffusion of media information (55). As digital information ecosystems can rapidly amplify perceived threats, the potential for discrimination driven by media is significantly heightened. Therefore, contextualizing existing scales to examine how specific media messages trigger discriminatory inclinations among the general population is of great importance. By adapting validated measures of discrimination to the unique context of the COVID-19 recovery phase, this study provides the empirical evidence from a public attitude perspective.

Parallel to discrimination, compliance with preventive measures serves as the primary behavioral outcome in health crisis management, being widely used in the medical field to assess patients' adherence to existing medical protocols (56). While early pandemic research emphasized the importance of increasing public compliance for risk management (57, 58) and the role of public trust in fostering compliance (59), more recent scholarship suggests a trust paradox or fatigue effect: high reliance on external governance can sometimes reduce individual vigilance, leading to a state of low self-efficacy. Besides, as the pandemic changing, compliance became less about passive adherence to rules (56) and more about active risk management driven by personal efficacy. A failure in this transition could exacerbate both the spread of disease and the intensification of discrimination (60).

This creates a theoretical tension. While elevating risk perception is essential for ensuring compliance (57), Stangl's framework suggests that this same heightened threat simultaneously acts as a driver for health discrimination. Although fear appeal literature has long grappled with this double-edged sword, empirical evidence remains insufficient in precisely differentiating how severity and susceptibility impact this trade-off (26). We propose that the resolution of this tension lies in the restoration of agency and a sense of control through perceived efficacy. Within the EPPM framework, self-efficacy and response efficacy serve as the psychological foundation for individuals to transition from fear control to danger control. As Smith et al. (61) noted, discrimination itself constitutes a severe social threat. When individuals confront a viral threat but lack sufficient efficacy, they could resort to discrimination as a defensive mechanism, projecting this social threat onto perceived risky others to regain a compensatory sense of psychological security (62, 63). Conversely, research confirms that the sense of control and agency inherent in self-efficacy fosters the volition and actual execution of proactive change (64, 65). In public health practice, enhancing audience awareness through mass media is a widely utilized anti-stigma strategy (66). Conceptually, disseminating clear guidelines on viral protection measures and their effectiveness emphasizes individuals' perceived control over barriers and stressors (67). This enhanced efficacy reduces negative emotional arousal—such as the anxiety, fear, and disgust associated with infection risks—thereby dismantling the psychological drive to endorse stigma, whether directed at others or internalized (68). Thus, efficacy acts as a cognitive stabilizer that decouples the perceived threat from the impulse to discriminate. Ultimately, we propose that efficacy serves as the key moderator capable of resolving the aforementioned tension: ensuring that heightened risk awareness leads to protective compliance rather than social exclusion.

**Figure 1:**
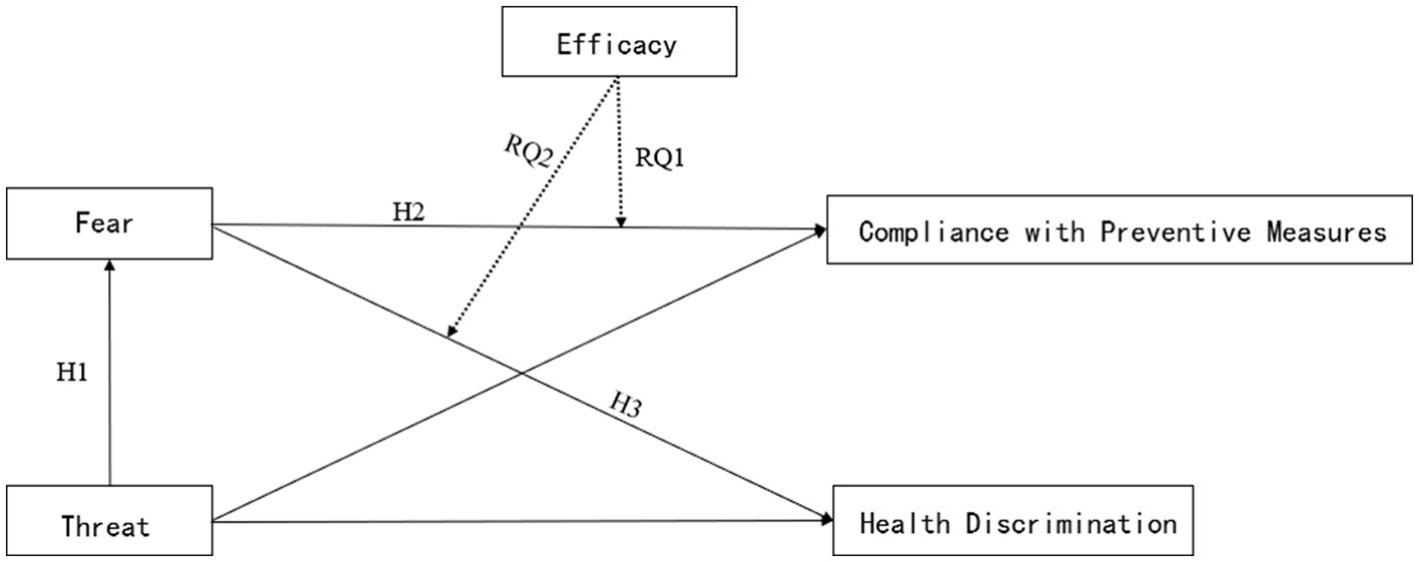
Research model.

Based on this, the study proposes the following hypotheses, research questions, and research model (see Figure 1):

H1: Threat information is positively correlated with the fear emotion.

H2: Fear emotions are positively correlated with compliance with preventive measures.

H3: Fear emotions are positively correlated with health discrimination.

RQ1: How does efficacy moderate the relationship between the fear emotion and compliance with preventive measures?

RQ2: How does efficacy moderate the relationship between the fear emotion and disease discrimination?

## 3 Method

### 3.1 Participants

This study used G^*^Power 3.1 to calculate the required sample size to ensure adequate statistical power. According to Cohen's (69) conventions, a medium effect size in multiple regression is defined as Cohen's *f*^2^ = 0.15. With the conventional parameters of α = 0.05 and a desired power of 0.95, the analysis indicated a required minimum sample size of 166 participants. Data collection took place from February 25th to 26th, 2023, during a period in China when the infection rates were declining after a peak, and there were adjustments to the pandemic prevention and control policies. We recruited 605 research participants online through the Credamo platform. Seven participants were excluded from the study due to failing the attention check questions, resulting in 598 valid participants. Among them, there were 299 males, with an average age of 30.64 years (SDage = 8.54). 428 participants reported having completed a university degree, and 435 participants reported living with older adults or young children.

Based on the two elements of EPPM (threat and efficacy), participants were randomly assigned to one of the conditions in a two (threat level: high vs. low) × 2 (efficacy level: high vs. low) between-subjects design. All participants volunteered to take part in the experiment, signed informed consent forms before the experiment began, and received a remuneration of six Chinese yuan after completing the experiment. This study was approved by the Ethics Committee of the Institutional Review Board of the School of Journalism and Communication (Tsinghua University No. TSJC 202301310005).

### 3.2 Procedure

Participants in the study first completed a survey to collect their demographic information and answered questions regarding their personal experiences with the COVID-19 pandemic. Questions included, for example, whether they had been infected with COVID-19, whether they were required to undergo centralized isolation due to experiencing COVID-19 symptoms or being in contact with confirmed or suspected cases of COVID-19, and whether they had changed, delayed, or canceled any of their medical services due to COVID-19.

Next, the participants were asked to read a news article. To manipulate threat (severity and susceptibility) and efficacy (self-efficacy and response efficacy), we collected and reorganized news reports to create four social media news posts presented with images and text. This material was attributed to a mainstream media source and focused on the latest expert opinions regarding the COVID-19 situation. It described the number of people affected by COVID-19, the rate of spread, preventive measures against COVID-19, and their effectiveness. Afterward, the participants completed questions regarding the fear emotion, perceived fear, perceived efficacy, compliance with preventive measures, and health discrimination.

### 3.3 Materials

Since the original scales were developed in English, we employed a translation and back-translation procedure involving two bilingual communication scholars to ensure linguistic and conceptual equivalence between the English and Chinese versions, while ensuring the items were contextually relevant to the current public health landscape in China.

#### 3.3.1 Fear

To measure the fear emotion, participants were asked to evaluate their current emotional state regarding COVID-19 by responding to three items assessing feelings of: (1) fearful, (2) afraid, and (3) anxious (adapted from Nabi & Myrick, 2019). Responses were rated on a scale from 1 (strongly disagree) to 7 (strongly agree; Cronbach's α = 0.932).

#### 3.3.2 Perceived threat

Participants rated their perceived threat using an adapted version of the Risk Behavior Diagnosis Scale (RBD Scale, Witte et al., 2001). Perceived threat consisted of susceptibility and severity, and participants responded using a scale from 1 (strongly disagree) to 7 (strongly agree). Susceptibility was measured with three items, such as “It is likely that I will get COVID-19,” while severity included three items, such as “I believe that COVID-19 is extremely harmful” (Cronbach's α = 0.944).

#### 3.3.3 Perceived efficacy

Assessment of perceived efficacy also came from an adapted version of the Risk Behavior Diagnosis Scale, including self-efficacy and response efficacy. Participants rated their responses on a scale from 1 (strongly disagree) to 7 (strongly agree). Self-efficacy consisted of three items, such as “I can easily comply with pandemic commands to prevent COVID-19,” while response efficacy also contained three items, such as “Complying with pandemic commands works in preventing COVID-19” (Cronbach's α = 0.940).

#### 3.3.4 Compliance with preventive measures

Participants completed a survey aimed at measuring their willingness to comply with preventive measures. They evaluated the necessity of following 12 preventive measures implemented by the government and mentioned in previous research (70–72) on a scale from 1 (not at all) to 7 (very much). Specific items included personal protective measures, such as “Wearing masks in public places,” adherence to government advice, such as “Leaving home only when allowed by the government,” and providing health advice to others, such as “Persuading high-risk family members (e.g., older adults over 80 years old) to get vaccinated and receive booster shots” (Cronbach's α = 0.971).

#### 3.3.5 Health discrimination

This scale included two subscales: stigma against COVID-19 patients and employment discrimination against COVID-19 survivors. The scale for stigma against COVID-19 patients was adapted from the COVID-19 Public Stigma Scale (COVID-PSS) (73) and consisted of 10 items, such as “Most COVID-19 patients do not take care of their health,” “Most COVID-19 patients are a burden to their families and society,” and “If I lived in a community with COVID-19 patients, I would fear getting infected with the virus.” Participants indicated their agreement with each statement using a seven-point scale, from 1 (strongly disagree) to 7 (strongly agree). The scale demonstrated good consistency (Cronbach's α = 0.966).

The employment discrimination scale for COVID-19 survivors was adapted from the Chinese version of the Zelaya HIV Stigma Scale (74) and the Anticipated Work Discrimination Scale (75). It included six statements related to employment discrimination against COVID-19 survivors, such as “Recovered COVID-19 patients should not be allowed to work alongside others” and “Recovered COVID-19 patients should not be employed as public school teachers.” Participants indicated their agreement with each statement on a scale from 1 (strongly disagree) to 7 (strongly agree; Cronbach's α = 0.968).

### 3.4 Pilot study

Prior to the main experiment, a pilot study was conducted to verify the effectiveness of the experimental manipulations. We recruited 120 participants, a sample size representing approximately 20% of the full study, which satisfies the recommended guidelines for pilot studies (76). Participants were randomly assigned to the four conditions. Independent samples *t*-tests confirmed the manipulation's success. Participants in the high-threat condition perceived significantly greater threat (*M* = 5.62, *SD* = 0.98) than those in the low-threat condition (*M* = 2.84, *SD* = 1.15), *t* (118) = 14.38, *p* < 0.001. Similarly, participants in the high-efficacy condition reported significantly higher perceived efficacy (*M* = 5.41, *SD* = 1.02) compared to the low-efficacy condition (*M* = 3.05, *SD* = 1.21), *t* (118) = 11.65, *p* < 0.001. These results confirmed that the stimuli effectively induced the intended psychological states.

## 4 Results

### 4.1 Reliability and validity

The reliability and validity of the measurement scales were examined using the full dataset (*N* = 598). Internal consistency was good, with Cronbach's α coefficients for all constructs ranging from 0.932 to 0.971. To validate the factor structure, we conducted a Confirmatory Factor Analysis (CFA) using AMOS. The five-factor measurement model (Fear, Perceived Threat, Perceived Efficacy, Health Discrimination, and Compliance) demonstrated a good fit to the data: *X*^2^/*df* = 2.68, *CFI* = 0.96, *TLI* = 0.95, RMSEA = 0.053 [90% CI [0.049, 0.057)], and *SRMR* = 0.041. All standardized factor loadings were statistically significant (*p* < 0.001) and ranged from 0.74 to 0.93, exceeding the recommended threshold of 0.50. Furthermore, the Composite Reliability (CR) values ranged from 0.93 to 0.97, and the Average Variance Extracted (AVE) values ranged from 0.68 to 0.82, indicating satisfactory convergent validity.

The demographic characteristics of the 598 participants are detailed in Table 1. In terms of education, a key characteristic of the sample is its higher level of education (87% holding a university degree), which is significantly higher than the national average of 15.5% from the 7th National Population Census (77). At the same time, the four experimental groups were relatively balanced and consistent across the dimensions of age, gender composition, education level, household income, employment status, and cohabitation status.

**Table 1:** Demographic characteristics of participants.

Characteristic	High threat × high efficacy (*n* = 150)	High threat × low efficacy (*n* = 150)	Low threat × high efficacy (*n* = 149)	Low threat × low efficacy (*n* = 149)	Total sample (*N* = 598)
Age (in years)					
Mean (SD)	31.18 (8.45)	31.23 (8.67)	30.61 (8.61)	29.53 (8.42)	30.64 (8.54)
Gender, *n* (%)					
Female	69 (46%)	83 (55%)	74 (50%)	73 (49%)	299 (50%)
Male	81 (54%)	67 (45%)	75 (50%)	76 (51%)	299 (50%)
Highest education, *n* (%)					
Senior high school or below	8 (5%)	6 (4%)	7 (5%)	6 (4%)	27 (5%)
Associate degree	9 (6%)	13 (9%)	14 (9%)	16 (11%)	52 (9%)
Bachelor's degree	106 (71%)	109 (73%)	107 (72%)	106 (71%)	428 (72%)
Master's degree or higher	27 (18%)	22 (14%)	21 (14%)	21 (14%)	91 (15%)
Monthly household income (CNY), *n* (%)					
Below ¥3,000	7 (5%)	6 (4%)	5 (3%)	9 (6%)	27 (5%)
¥3,000–5,000	16 (11%)	7 (5%)	14 (9%)	15 (10%)	52 (9%)
¥5,001–10,000	56 (37%)	48 (32%)	47 (32%)	48 (32%)	199 (33%)
¥10,001–20,000	39 (26%)	60 (40%)	60 (40%)	54 (36%)	213 (36%)
Above ¥20,000	32 (21%)	29 (19%)	23 (16%)	23 (16%)	107 (18%)
Employment status, *n* (%)					
Student	29 (19%)	32 (21%)	38 (26%)	35 (23%)	134 (22%)
Employed	119 (79%)	116 (77%)	109 (73%)	114 (77%)	458 (77%)
Unemployed	1 (1%)	0 (0%)	1 (1%)	0 (0%)	2 (0%)
Retired/unable to work	1 (1%)	2 (2%)	1 (0%)	0 (0%)	4 (1%)
Cohabitation with older adults or children, *n* (%)					
Yes	116 (77%)	114 (76%)	103 (69%)	102 (68%)	435 (73%)
No	34 (23%)	36 (24%)	46 (31%)	47 (32%)	163 (27%)
Political attitude (1–7 scale)					
Mean (SD)	4.9 (1.27)	4.91 (1.15)	4.99 (1.10)	4.83 (1.17)	4.90 (1.17)

### 4.2 Correlation and regression analyses

To test our hypotheses, we first conducted a Pearson correlation analysis to explore the preliminary associations between key variables (see Table 2). We acknowledge the reviewer's observation that the magnitudes of the correlation coefficients are in the small to moderate range. For example, the correlation between threat and compliance with preventive measures was significant but small (*r* = 0.158, *p* < 0.01), while the correlation between fear and health discrimination was moderate (*r* = 0.377, *p* < 0.01). The statistical significance of these relationships is robust due to our large sample size, indicating they are reliable and not a product of random chance.

**Table 2:** Pearson correlation analysis.

Variables	Efficacy	Threat	Fear	Compliance with preventive measures	Health discrimination
Efficacy	1				
Threat	0.000	1			
Fear	−0.094^*^	0.214^**^	1		
Compliance with preventive measures	−0.061	0.158^**^	0.199^**^	1	
Health discrimination	0.030	0.262^**^	0.377^**^	0.162^**^	1

^*^p < 0.05;

^**^p < 0.01.

To more rigorously test our hypotheses and ascertain the unique predictive power of threat and fear, we proceeded with a series of multiple linear regression analyses, controlling for key demographic variables (see Table 3). This method allows us to see if the effects of threat and fear persist after accounting for confounding factors.

**Table 3:** Regression models.

Predictors	Model 1	Model 2	Model 3
	Fear	Compliance with preventive measures	Health discrimination
Gender	0.014 (0.106)	0.272 (1.598)	−0.343^**^ (−2.795)
Age	−0.025^*^ (−2.463)	0.013 (1.073)	−0.012 (−1.299)
Highest academic degree	−0.071 (−0.722)	−0.242 (−1.963)	−0.010 (−0.116)
Household monthly income	−0.148^*^ (−2.117)	−0.089 (−1.013)	−0.108 (−1.706)
Employment status	0.288 (1.751)	−0.155 (−0.753)	−0.032 (−0.214)
Co-residence with family	0.157 (0.971)	−0.224 (−1.107)	0.417^**^ (2.859)
Political attitude	−0.106 (−1.825)	−0.134 (−1.833)	0.009 (0.177)
Threat	0.738^***^ (5.455)	0.551^**^ (3.180)	0.631^***^ (5.047)
Fear		0.202^***^ (3.920)	0.315^***^ (8.470)
*R* ^2^	0.077	0.079	0.205
Adjusted *R*^2^	0.064	0.065	0.193
*F*-statistic	6.113^***^	5.634^***^	16.817^***^

^*^p < 0.05;

^**^p < 0.01;

^***^p < 0.001.

The results strongly supported our hypotheses. Predicting Fear (Model 1): the threat condition remained a powerful and significant positive predictor of fear (*B* = 0.738, *t* = 5.455, *p* < 0.001), confirming Hypothesis 1. Predicting Compliance (Model 2): crucially, even when tested together, both the threat condition (*B* = 0.551, *t* = 3.180, *p* < 0.01) and fear emotion (*B* = 0.202, *t* = 3.920, *p* < 0.001) independently predicted compliance with preventive measures. This confirms Hypothesis 2 and demonstrates their distinct contributions. Predicting Discrimination (Model 3): similarly, both the threat condition (*B* = 0.631, *t* = 5.047, *p* < 0.001) and fear emotion (*B* = 0.315, *t* = 8.470, *p* < 0.001) were highly significant positive predictors of health discrimination, supporting Hypothesis 3. Notably, the model explained a substantial portion of the variance in health discrimination (Adjusted *R*^2^ = 0.193).

In conclusion, while the simple bivariate correlations were modest, the regression analyses provide robust evidence. They demonstrate that threat and fear are significant and independent drivers of both compliance and health discrimination, even when controlling for demographic factors. This confirms their theoretical and practical importance beyond the initial correlation magnitudes.

### 4.3 Mediation analysis

In this study, the Bootstrap method with the PROCESS macro program in SPSS was employed to examine the serial mediation effects. A sample size of 5,000 was set (typically recommended to be above 1,000). The confidence level for the intervals was set at 95% (commonly set at 90%, 95%, and 99%). Biased-corrected confidence intervals were used to observe their upper and lower bounds. If the biased-corrected confidence interval of the indirect effect does not include zero, it indicates the presence of a mediation effect.

Regarding the mediation pathway of “Threat Group ⇒ Fear Emotion ⇒ Health Discrimination,” the indirect effect value is 0.232, and the 95% interval does not include zero (95% CI: 0.133~0.345), indicating the establishment of the mediation effect of fear emotion in the relationship between the threat group and health discrimination. Similarly, considering the mediation pathway of “Threat Group ⇒ Fear Emotion ⇒ Compliance with Preventive Measures,” the indirect effect value is 0.149, and the 95% interval does not include zero (95% CI: 0.060~0.254), suggesting the presence of the mediation effect of fear emotion in the relationship between the threat group and compliance with preventive measures.

### 4.4 Moderation analysis

To test our hypotheses regarding moderated mediation, we utilized the PROCESS macro for SPSS (Model 7), with efficacy serving as the moderator of the mediation path from threat to behavioral outcomes via fear. This analysis allows us to first examine the moderation effect and then the conditional indirect effects (see Table 4).

**Table 4:** Moderated-mediation analysis.

Path	Efficacy	Effect	BootSE	BootLLCI	BootULCI
Threat ⇒ fear ⇒ health discrimination	Low	0.355	0.072	0.221	0.506
	High	0.137	0.053	0.045	0.255
Threat ⇒ fear ⇒ with preventive measures	Low	0.030	0.061	−0.093	0.150
	High	0.241	0.069	0.117	0.384

#### 4.4.1 Moderation on the fear → health discrimination path

First, we examined the interaction effect on health discrimination. The regression results revealed a significant negative interaction between fear and efficacy (*B* = −0.252, *t* = −4.083, *p* < 0.001). This negative coefficient indicates that efficacy weakens the positive relationship between fear and health discrimination.

**Figure 2:**
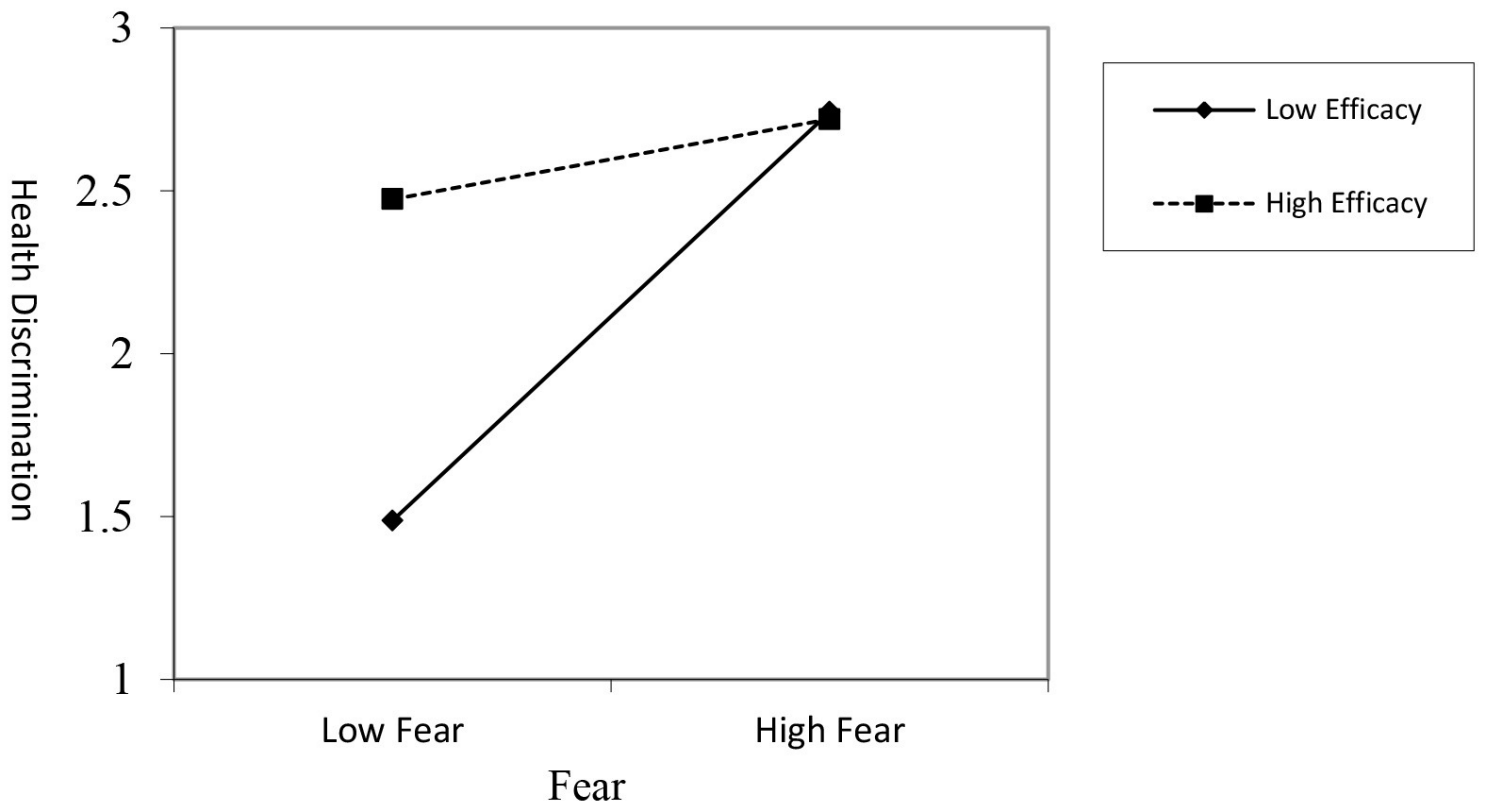
The moderating effect of efficacy on fear-induced health discrimination.

This interaction is visualized in Figure 2. The simple slopes analysis shows that while fear's positive effect on health discrimination is significant at both high and low levels of efficacy, the effect is substantially stronger for individuals with low efficacy compared to those with high efficacy.

Consistent with this, the analysis of the conditional indirect effect (see Table 5) confirms this pattern. The indirect effect of threat on health discrimination (via fear) was significant but much stronger under the condition of low efficacy [Effect = 0.355, 95% CI (0.221, 0.506)] than under high efficacy [Effect = 0.137, 95% CI (0.045, 0.255)].

**Table 5:** Mediation analysis.

Path	Effect	Boot SE	BootLLCI	BootULCI
Threat ⇒ fear ⇒ health discrimination	0.232	0.053	0.133	0.345
Treat ⇒ fear ⇒ compliance with preventive measures	0.149	0.050	0.060	0.254

#### 4.4.2 Moderation on the fear → compliance with preventive measures path

**Figure 3:**
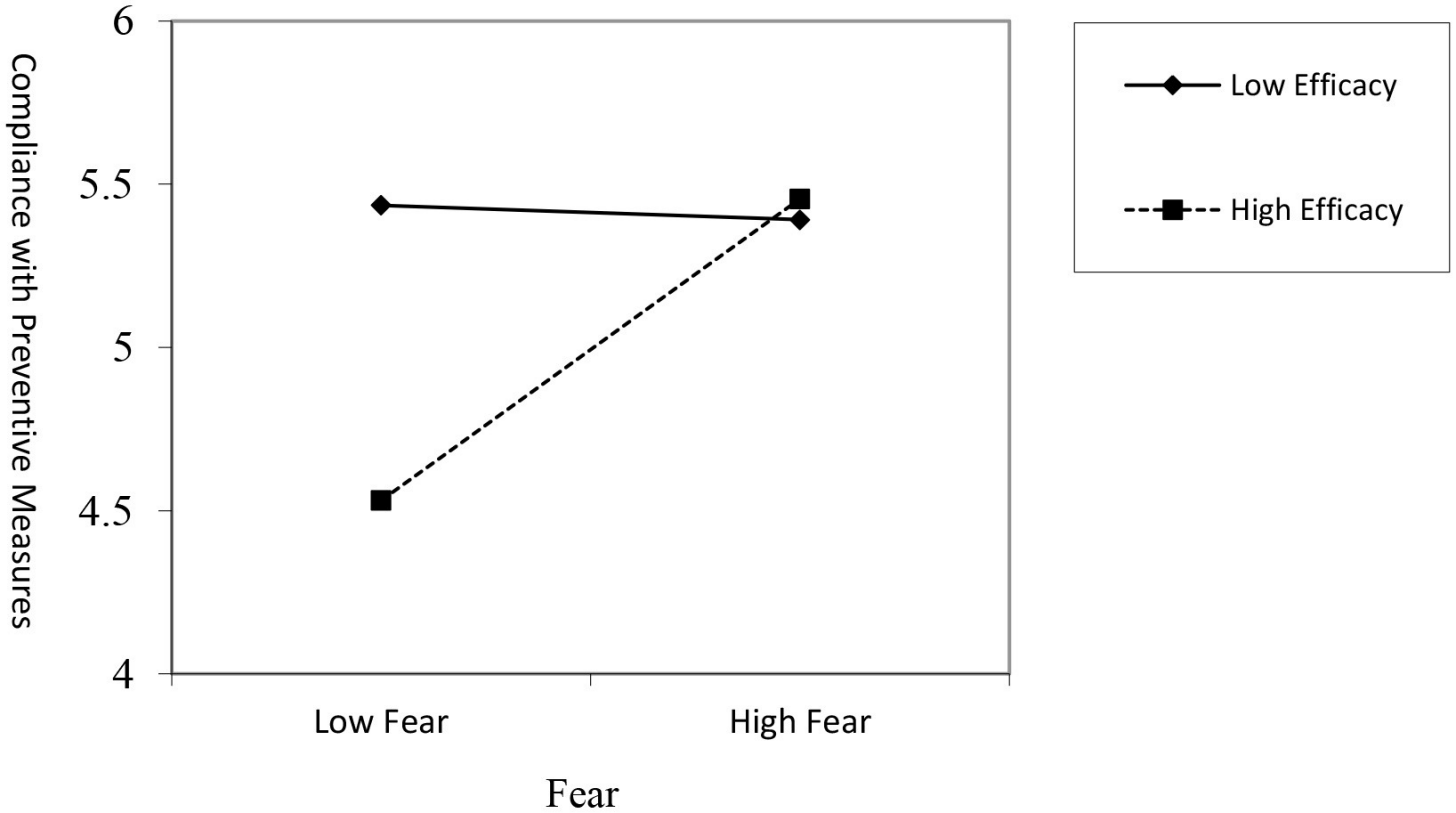
The moderating effect of efficacy on fear-induced compliance with preventive measures.

Next, we examined the interaction effect on compliance. The results showed a significant positive interaction between fear and efficacy (*B* = 0.242, *t* = 2.833, *p* < 0.01). This positive coefficient signifies a positive moderation effect, meaning that higher levels of efficacy strengthen the positive relationship between fear and compliance. This interaction is clearly visualized in Figure 3. The simple slopes plot demonstrates that the positive relationship between fear and compliance is substantially stronger and steeper for individuals with high efficacy. In contrast, for those with low efficacy, this relationship becomes flat and non-significant. This confirms that efficacy functions as a crucial catalyst, enabling fear to be channeled into adaptive actions like compliance.

The conditional indirect effect analysis (Table 5) provides a clear explanation. For the path “Threat → Fear → Compliance,” the indirect effect was significant and strong only under the condition of high efficacy [Effect = 0.241, 95% CI (0.117, 0.384)]. In contrast, for individuals with low efficacy, this indirect effect was not statistically significant [Effect = 0.030, 95% CI (−0.093, 0.150)], as the confidence interval contains zero. This supports our hypothesis that efficacy is a crucial condition for fear to be channeled into positive preventive actions.

## 5 General discussion

**Figure 4:**
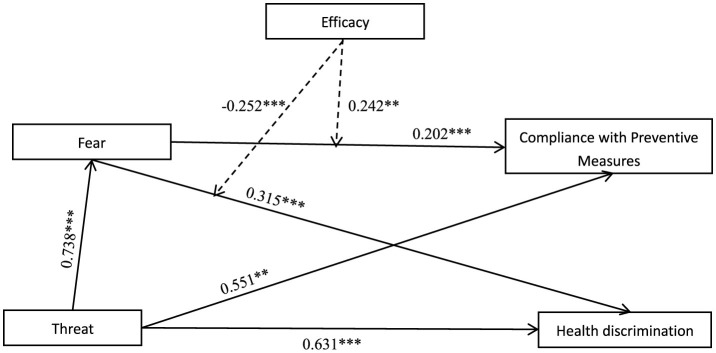
Research result model. ^**, ***^indicates that the path coefficient is statistically significant at the *p* < 0.01, *p* < 0.001 level.

The COVID-19 pandemic has not only posed a profound threat to global public health but has also deeply impacted social norms and interactions. In the environment fraught with uncertainty, health communication, particularly through news media, plays a critical role in promoting public compliance with protective measures. Fear appeals are a common strategy in this context, intended to highlight the risks and motivate preventive action (78). However, such appeals can potentially trigger unintended negative consequences like social discrimination. The present study, guided by the Extended Parallel Process Model (EPPM), aimed to explore these complex effects, examining how news messages conveying threat and efficacy influence both compliance with preventive measures and health discrimination (see Figure 4).

Our findings first affirmed the powerful, yet conflicting, role of threat-induced fear. Consistent with our hypotheses, news messages framed with high threat were a significant predictor of fear, which in turn was associated with two divergent outcomes. On one hand, fear correlated positively with a greater willingness to comply with preventive measures, underscoring its role in motivating protective behaviors (79). On the other hand, this same emotion was also positively correlated with greater health discrimination. This establishes a critical “double-edged sword” dilemma for health communicators.

Furthermore, we sought to explore the role of efficacy in moderating these pathways. A compelling pattern emerged from our exploratory analysis. When high-threat information was paired with high efficacy, the positive relationship between fear and compliance was significantly strengthened, appearing to effectively channel motivation toward preventive measures. This finding aligns with the core danger control tenets of the EPPM. Conversely, our analysis also suggests that high efficacy may buffer against the negative consequences of fear; under high efficacy conditions, the fear's promotion of health discrimination appeared to be suppressed.

EPPM-based studies have primarily focused on strategies to promote health behaviors in everyday situations, and research on health protection during public health emergencies has gained increased attention since COVID-19 (35, 80). While the moderating role of efficacy on behavioral compliance aligns with established meta-analytic evidence (17, 37, 81), our study offers a distinct theoretical contribution by extending the EPPM's scope from individual behavioral adoption to interpersonal social outcomes. Previous EPPM scholarship has pre-dominantly focused on how efficacy determines the shift between danger control and fear control regarding personal protection. However, our findings reveal that these psychological processes also spill over into social cognition. Specifically, we identified that low efficacy does not merely lead to defensive avoidance, but can also manifest externally as health discrimination against victims. This suggests that stigmatization may serve as a maladaptive“fear control”mechanism in a social context. By demonstrating that high efficacy can buffer against this process of social stigmatization, our study bridges the gap between fear appeal theories and the stigma literature, answering calls to examine the unintended negative consequences of threatening health messages (26).

As we transition into the post-pandemic era, the mechanism identified in this study—where fear unaccompanied by efficacy triggers social discrimination—remains highly relevant for managing other infectious diseases, such as seasonal influenza and Mpox. Theoretically, this suggests that the EPPM should be reconceptualized not merely as a model predicting individual behavioral adoption, but as a framework for managing collective social sentiment. Our study contributes to risk communication by demonstrating that efficacy beliefs act as a critical buffer against the “social externalization” of fear control—specifically, discrimination. From a practical standpoint, these findings update the ethical mandate for health communication and offer actionable guidance for news media: the effectiveness of communication must be measured not only by the adoption of preventive behaviors but also by the minimization of unintended social discrimination. To foster sustained public cooperation while preserving social solidarity, communication campaigns must emphasize clear, actionable steps and bolster the public's confidence in their ability to perform them. This balanced approach appears to be the most promising path for channeling fear productively, maximizing protection while minimizing pre-judice. Thus, within health and science communication, empowering the public with high-efficacy solutions is not just a strategic necessity for compliance, but an ethical obligation to maintain social cohesion during public health crises.

## 6 Limitations and future research

This study presents an exploration of the potential contribution of the EPPM in promoting compliance with preventive measures during sudden public health crises and reducing health discrimination. However, several limitations should be considered. Firstly, the study did not limit the infection status of the participants, making it challenging to detect differences in discriminatory attitudes between infected and uninfected individuals. Future research could explore this comparison to provide deeper insights. Secondly, while our model explained nearly 20% of the variance in health discrimination, which is noteworthy in a crisis context, the low *R*^2^ values for other outcomes indicate that a large portion of variance remains unexplained. Future research could build more comprehensive models by incorporating other variables, such as media consumption patterns, social trust, and individual psychological traits.

Furthermore, this study was conducted within a single, pre-dominantly collectivist cultural context. Future research should examine these dynamics cross-culturally. For instance, comparing how collectivist vs. individualistic societies (82) respond to public health messaging that balances threat and efficacy could yield valuable insights. Besides, while our study did not focus on racial or ethnic identity, it is crucial to acknowledge that the COVID-19 pandemic was heavily racialized on a global scale (83, 84). This global discourse may have influenced in-group and out-group attitudes even within a more ethnically homogeneous context. Future studies could incorporate measures of ethnic identity and perceived discrimination to explore these dynamics.

Finally, a notable finding was that compliance persisted even under low-efficacy conditions. This phenomenon may be attributed to the unique sociocultural context of the study period: the high level of environmental uncertainty, coupled with long-established governmental norms, likely fostered a form of behavioral inertia. Such behavior reflects the defensive inaction theorized by Witte (13), suggesting that individuals may adhere to preventive measures out of social habituation or normative pressure rather than autonomous agency. Furthermore, while this study substantiates the role of individual self-efficacy in mitigating health discrimination, the conceptualization of self-efficacy itself is culturally contingent. For instance, in collectivist settings (85) and among specific demographics such as female immigrants (86), self-efficacy beliefs can be more other-oriented. This also suggests that collective efficacy could be a vital construct in public health crises with the potential to significantly expand the EPPM (61, 87). Future research should investigate the synergy between individual and collective efficacy. By exploring how these dual levels of belief collectively provide a psychological buffer, future studies can build on these insights to offer a more comprehensive and contextualized framework for reducing discrimination of health risks, fostering social cohesion, and understanding health behavior in crisis settings.

## Data Availability

The datasets presented in this study can be found in online repositories. The names of the repository/repositories and accession number(s) can be found below: the datasets generated during and/or analyzed during the current study are available in the HARVARD Dataverse repository, (https://doi.org/10.7910/DVN/63ISTM).
