# Supplementary material for: Threat, efficacy, and the ambivalent role of COVID-19 news: an EPPM analysis of health discrimination and preventive behaviors
Source: Front Public Health. 2026 Apr 2;14:1790676. doi: 10.3389/fpubh.2026.1790676 (PMC13083066; doi:10.3389/fpubh.2026.1790676)
Supplement: Supplementary file 1 [file Table_1.docx]

| Title | | COVID-19 Pandemic: Latest Expert Analysis Released | |
| --- | --- | --- | --- |
| Threat |  | High | Low |
|  | Susceptibilit  y | On Tuesday, the head of the National Health Commission responded, stating that the total number of new locally transmitted cases has exceeded 30,000 in the past ten days. China is entering a high-level platform period of the pandemic. Currently, the prevalent Omicron variant, strain BF.7, in the country can infect others within 24 hours of contracting the virus. Chinese CDC experts predict that eventually, over 80%-90% of the population will experience infection. | On Tuesday, the head of the National Health Commission responded, stating that the number of daily new locally transmitted cases has been continuously decreasing for six days, indicating an overall improvement in the COVID-19 epidemic prevention and control situation. Currently, the prevalent Omicron variant, strain BF.7, in the country, although highly transmissible, exhibits very low pathogenicity, with the majority of infected individuals being asymptomatic. |
|  | Severity | Zhong Nanshan stated that 22% of individuals may continue to encounter challenges in returning to their regular work routines even after recovering from the illness. Once contracted, the Omicron variant could result in severe health risks and long-term consequences, such as headaches, attention disorders, and respiratory issues. Elderly individuals face higher susceptibility to fatal outcomes, whereas young and middle-aged adults might experience symptoms like fatigue and sleep disorders following infection.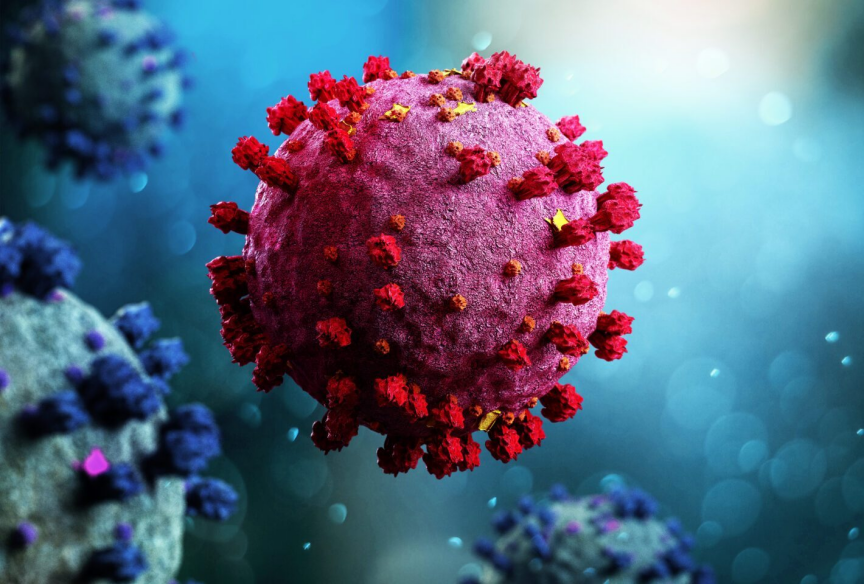 | Zhong Nanshan stated that Omicron infection is primarily characterized by asymptomatic cases, where individuals may be infected but exhibit no noticeable symptoms or discomfort. On December 5, 2022, American virologists conveyed that when solely considering deaths directly attributed to COVID-19, the infection fatality rate of the virus is approximately 0.04%, a figure comparable to that of the seasonal flu.  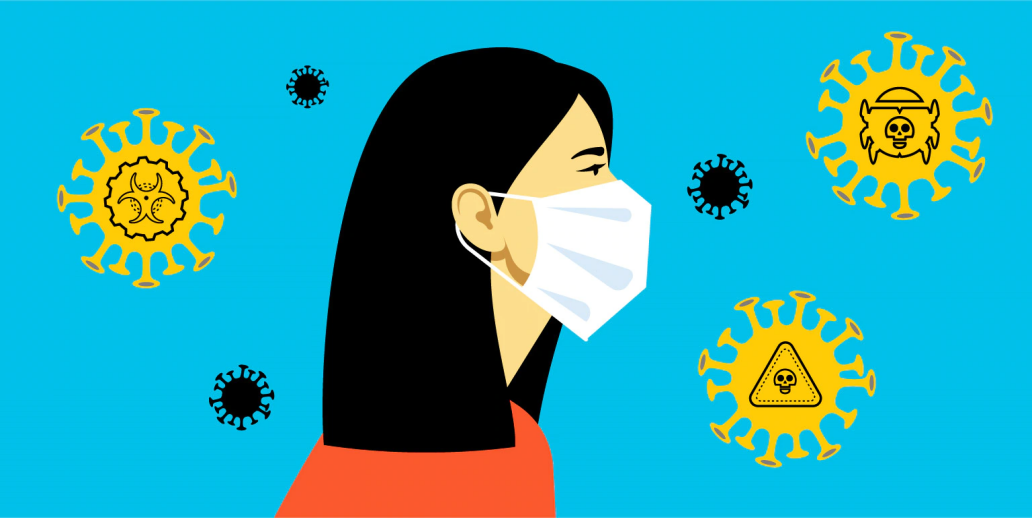 |

| Efficacy |  | High | Low |
| --- | --- | --- | --- |
|  | Response efficacy | Vaccination has considerably decreased the severity and mortality rate of the disease. In daily life, wearing standard medical masks can effectively reduce the likelihood of infection by 80%. On one hand, the human immune system remains actively engaged in combating COVID-19, while on the other hand, medications like Lianhua Qingwen can alleviate discomfort in individuals who have tested positive for the virus. The future trajectory of the COVID-19 virus may mirror that of seasonal influenza, necessitating regular updates to COVID-19 vaccines, similar to influenza vaccines. | Our nation has entered a phase of widespread infections, and due to the discontinuation of nucleic acid testing, some positive patients may be observed in public settings. Furthermore, the virus can spread through contaminated surfaces, the air, and water pipes, making it possible to contract the virus even while wearing masks. Moreover, the efficacy of vaccines declines over time, and although medications like Lianhua Qingwen can alleviate symptoms, they cannot expedite the transition from a positive to negative status for infected individuals. |
|  | self-efficacy | Overall, proactively getting vaccinated and taking personal protective measures are the best strategies to cope with the uncertain future of viral evolution. Currently, China has achieved nationwide free vaccination against the COVID-19 virus. Even if infected with COVID-19, the majority of patients experience mild symptoms, and 99% of them can fully recover within 7 to 10 days through home treatment, while young individuals usually require only 3 to 4 days. Professors from the Third Hospital of Central South University have stated that recovered individuals develop resistance to the virus and, in a short period of time, are unlikely to be reinfected by the same strain.  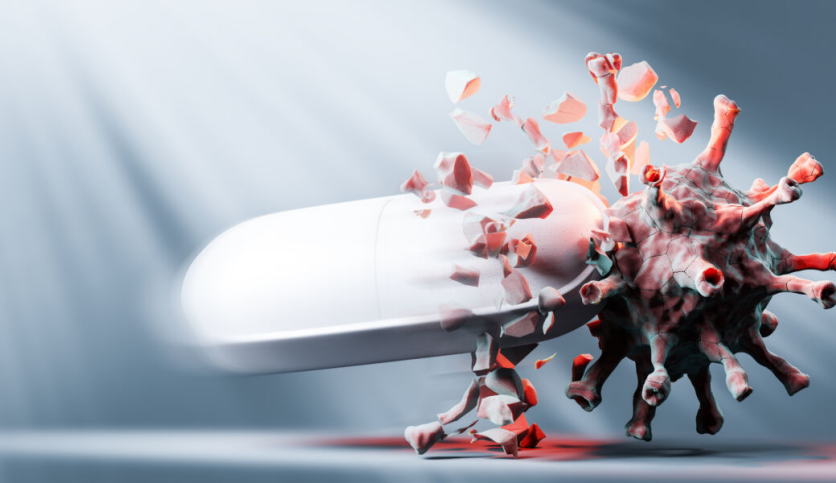 | Overall, in the face of uncertain viral evolution, existing protective measures have limited effectiveness. The Omicron variant, strain BF.7, exhibits strong transmissibility and rapid spread, making regular measures like wearing masks and using disinfectants insufficient to completely eliminate the strain. Compared to previous variants like Delta, the Omicron variant is more prone to causing reinfections, making lifelong immunity unattainable. Professors from the Third Hospital of Central South University have stated that 10% of patients in the United States experienced a second infection, highlighting the notion that "early exposure leads to lifelong immunity" as unfeasible.  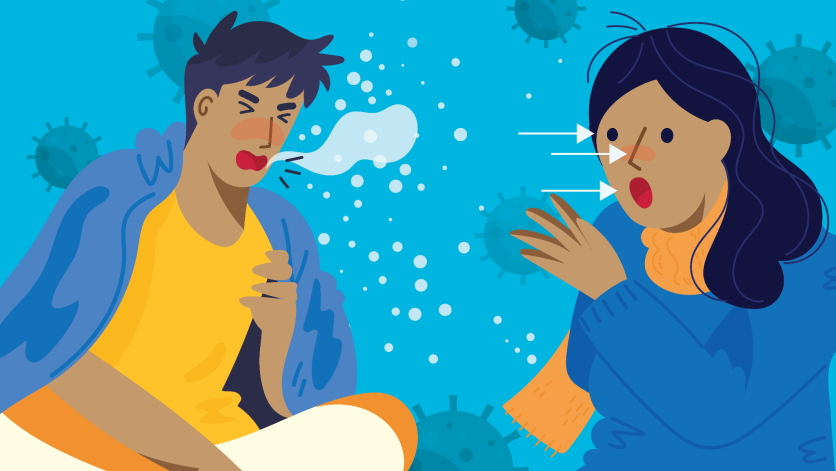 |
